# ﻿Two new species of *Miersia* and their phylogenetic placements alongside the recently described *M.putaendensis* (Gilliesieae, Allioideae, Amaryllidaceae)

**DOI:** 10.3897/phytokeys.211.87842

**Published:** 2022-10-20

**Authors:** Nicolás García, Claudia Cuevas, Joaquín E. Sepúlveda, Arón Cádiz-Véliz, María José Román

**Affiliations:** 1 Herbario EIF & Laboratorio de Evolución y Sistemática, Facultad de Ciencias Forestales y de la Conservación de la Naturaleza, Universidad de Chile, Av. Santa Rosa 11315, La Pintana, Santiago, Chile Universidad de Chile Santiago Chile; 2 Instituto Agroecosistemas, Curicó, Chile Instituto Agroecosistemas Curicó Chile; 3 Instituto de Biología, Facultad de Ciencias, Pontificia Universidad Católica de Valparaíso, Campus Curauma, Avenida Universidad 330, Valparaíso, Chile Pontificia Universidad Católica de Valparaíso Valparaíso Chile; 4 Department of Biology, University of Florida, Gainesville, Florida 32611, USA University of Florida Gainesville United States of America

**Keywords:** Alliaceae, Central Chile, IUCN, phylogeny, taxonomy

## Abstract

Two new species of the Chilean endemic genus *Miersia* (Gilliesieae, Allioideae, Amaryllidaceae) are described, *M.stellata* and *M.raucoana*, alongside morphological descriptions, a distribution map, illustrations, conservation status assessments, and an updated key to all species of *Miersia*. Additionally, phylogenetic analyses of DNA sequences were performed to inquire into the evolutionary affinities of both new species and the recently described, *M.putaendensis*, within the tribe Gilliesieae.

## ﻿Introduction

Amaryllidaceae J.St.-Hil. consists of three subfamilies in its modern circumscription: Amaryllidoideae Burnett, Agapanthoideae Endl., and Allioideae Herb. (Chase et al. 2009; Pellicer et al. 2017). Allioideae includes several bulbous or rhizomatous species of agricultural and ornamental importance divided into four tribes: Allieae Dumort is distributed in the Northern Hemisphere, Tulbaghieae Endl. ex Meisn. is restricted to South Africa, and Leucocoryneae Ravenna and Gilliesieae Baker, are both endemic to South America (Sassone and Giussani 2018; Escobar et al. 2020). Recent phylogenetic and karyotypic studies have determined that the crown group of Allioideae diversified ~62 Mya, and support a Gondwanan origin for Allioideae, with vicariant events as the cause of the intercontinental distribution of its four tribes (Costa et al. 2020).

Gilliesieae is a poorly known tribe comprising several threatened species (Torres-Mellado et al. 2012) characterized by zygomorphic flowers, a character state that is divergent from the rest of Allioideae, which typically have actinomorphic flowers (Rudall et al. 2002; Escobar et al. 2020; García et al. 2022). This tribe is composed of eight genera mainly distributed in the southern cone of South America: *Ancrumia* Harv. ex Baker, *Gethyum* Phil., *Gilliesia* Lindl., *Miersia* Lindl., *Speea* Loes. (Mediterranean Chile), *Solaria* Phil. (Chile and Argentina), *Trichlora* Baker (Peru) and *Schickendantziella* Speg. (Argentina and Bolivia) (Escobar 2012; Escobar et al. 2012). Phylogenetic studies of Gilliesieae suggested that its most recent common ancestor diverged during the Miocene ca. (29-) 18 (-7) Mya, a period characterized by a global increase in temperature, a retreat of glacial cover and great tectonic activity that produced the uplift of the Andes, a process directly related to the diversification of this clade (Sassone and Giussani 2018; Costa et. al 2020).

*Miersia* is endemic to central Chile and includes bulbous herbs with zygomorphic flowers, perigones formed by six free green-violaceous tepals, sometimes very reduced tepaliferous appendages, and in most species, a staminal tube formed by the fusion of 6 fertile stamens (Rudall et al. 2002; Escobar 2012; Cádiz-Véliz 2021). The latest taxonomic and phylogenetic treatment of *Miersia* comprised five accepted species and evidenced that the single species in *Speea*, *S.humilis* (Phil.) Loes. ex K.Krause, is embedded within *Miersia* (Escobar 2012; Escobar et al. 2020). Recently, Cádiz-Véliz (2021) described the new species *M.putaendensis* A.Cádiz-Véliz from the Valparaíso Region based on its morphological distinctiveness; however, its phylogenetic position is still unknown within *Miersia*.

As the result of two independent field explorations in central Chile during the winter (August) of 2020, two undescribed species of *Miersia* were discovered. The first species was found on a rocky outcrop near the town of Lampa (33°16'S, 70°51'W, 600 m a.s.l.), Metropolitan Region of Santiago, and the second, inhabiting a rocky slope close to the town of Rauco, Curicó (34°54'S, 71°22'W, 535 m a.s.l.), Maule Region. This study describes these new species and provides a distribution map, illustrations and conservation assessments for them, besides an updated identification key to all species of *Miersia*. Additionally, the recently described *Miersiaputaendensis* and both species described here were placed in the phylogeny of Gilliesieae to evaluate their evolutionary affinities within this tribe.

## ﻿Methods

### ﻿Herbarium and fieldwork

**Figure 1.**
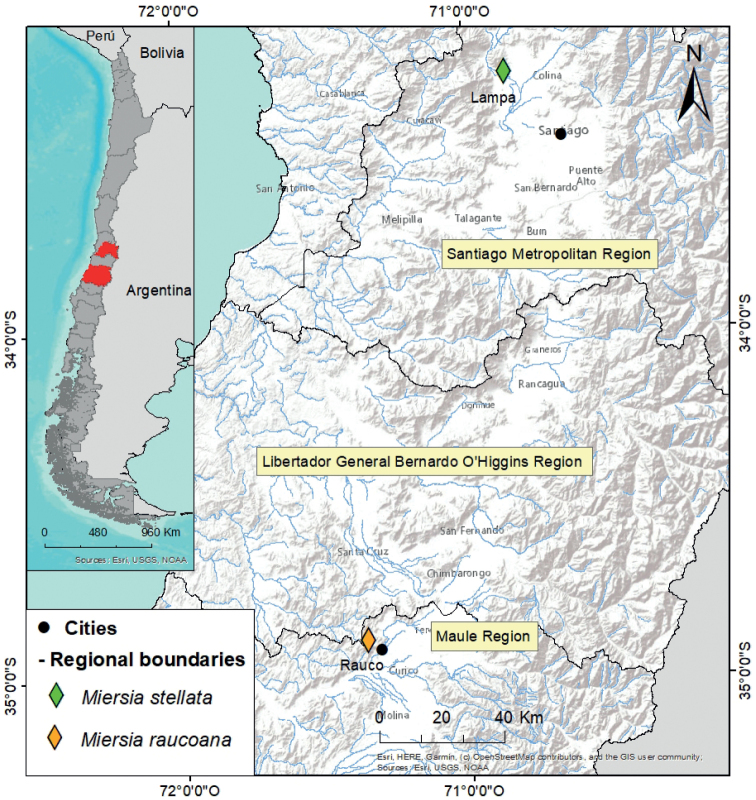
Distribution of *Miersiastellata* (green diamond) and *Miersiaraucoana* (orange diamond). Map by Claudia Cuevas.

Fieldwork to collect the type specimens and silica-dried leaves for DNA extractions was carried out in June 2021 in Cerro Quilhuica, Lampa, and in July 2021 in the hills of Rauco, Curico (Fig. 1). Specimens were collected and deposited in the collections of the EIF, CONC, JBN, and SGO herbaria (Thiers 2022, updated continuously). Additionally, flowers were collected in 70% ethanol for morphological measurements and descriptions. The main taxonomic literature on Gilliesieae was consulted for morphological descriptions of previously described species (Ravenna 2000; Escobar et al. 2010, 2020; Escobar 2012; Cádiz-Véliz 2021). Plant terminology follows Beentje (2012). Measurements were made using a Motic MZ-171 stereomicroscope for structures smaller than 1 cm or with the naked eye for larger structures. All widths were measured over the widest portion of the structure.

### ﻿Taxon sampling and phylogenetic analyses

Genomic DNA was extracted from type specimens of the three species of *Miersia* (*Miersiaputaendensis: A. Cádiz-Véliz 548*, EIF 14041, isotype; *Miersiaraucoana: N. García* et al. *6139*, EIF 14824, holotype; *Miersiastellata: N. García & C. Cuevas 6132*, EIF 14823, holotype) using the Qiagen DNeasy Plant Mini Kit (Qiagen, Hilden, Germany) following the manufacturer’s instructions. Based on previous studies and sequences available for Gilliesieae (Escobar et al. 2020), we amplified the *rbcL* gene and *trnL-F* intron and spacer, which together form our chloroplast DNA (cpDNA) matrix, and the nuclear ribosomal DNA internal transcribed spacer (nrITS). The amplification of DNA fragments followed the protocols described by Escobar et al. (2020). Sequencing was performed using the same amplification primers, by Macrogen, South Korea. We generated nine new sequences and deposited them in GenBank (Suppl. material 3: Table S1); the remaining sequences were obtained from datasets by Escobar et al. (2020), available in the Treebase repository (N 26352) and in Zenodo (doi: 10.5281/zenodo.6581791).

Preliminary examination of all sequence data revealed that several were identical; therefore, we kept a single sequence per species in general (two accessions for *Gethyumatropurpureum* Phil. and *Solariamiersioides* Phil.) and excluded accessions that were represented in a single locus dataset. Herbarium material of the accession Escobar 84 (CONC, EIF), which was treated as *Miersia* sp. by Escobar et al. (2020), was reassessed and is treated as Miersiacf.chilensis Lindl. in the present study. Hence, our taxon sampling was identical and complete for all loci (only *rbcL* missing from *Miersiacornuta* Phil. 152) and included 20 ingroup (Gilliesieae) and 6 outgroup accessions (Suppl. material 3: Table S1), that correspond to five Leucocoryneae taxa and one representative of tribe Tulbaghieae (*Tulbaghiacapensis* L.). Editing and assembling of sequences were performed in Geneious Prime v.2022.1.1 (https://www.geneious.com). Sequences were aligned using MAFFT v.1.4.0 (Katoh and Standley 2013).

A maximum likelihood (ML) analysis was performed for the concatenated matrix of all loci using RAxML-NG v.1.1.0 (Kozlov et al. 2019), GTR+Γ as the model of molecular evolution, and conducting 100 tree searches using 50 random and 50 parsimony-based starting trees to pick the best-scoring topology. Each cpDNA locus (*rbcL*, *trnL-F*) and nrITS were considered as separate partitions to increase model fit by accommodating locus-specific variation. Subsequently, likelihood bootstrap analyses (Felsenstein 1985) were conducted with 1,000 pseudoreplicates. Additionally, we performed separate analyses for the nrITS and cpDNA datasets following the same parameters as above, except for the tree searches which were performed using 25 random and 25 parsimony-based starting trees for each. All trees were rooted using *Tulbaghiacapensis*, following Escobar et al. (2020). Alignments of cpDNA and nrITS matrices, as well as maximum likelihood and bootstrap trees, are available in Zenodo (doi: 10.5281/zenodo.6581791).

### ﻿Conservation assessment

The assessment of the conservation status of both species was conducted using the International Union for Conservation of Nature (IUCN 2017) criteria. The extent of occurrence (EOO) and area of occupancy (AOO) were calculated using Google Earth and threats were identified from field observations.

## ﻿Results

### ﻿Phylogenetic analyses

**Figure 2.**
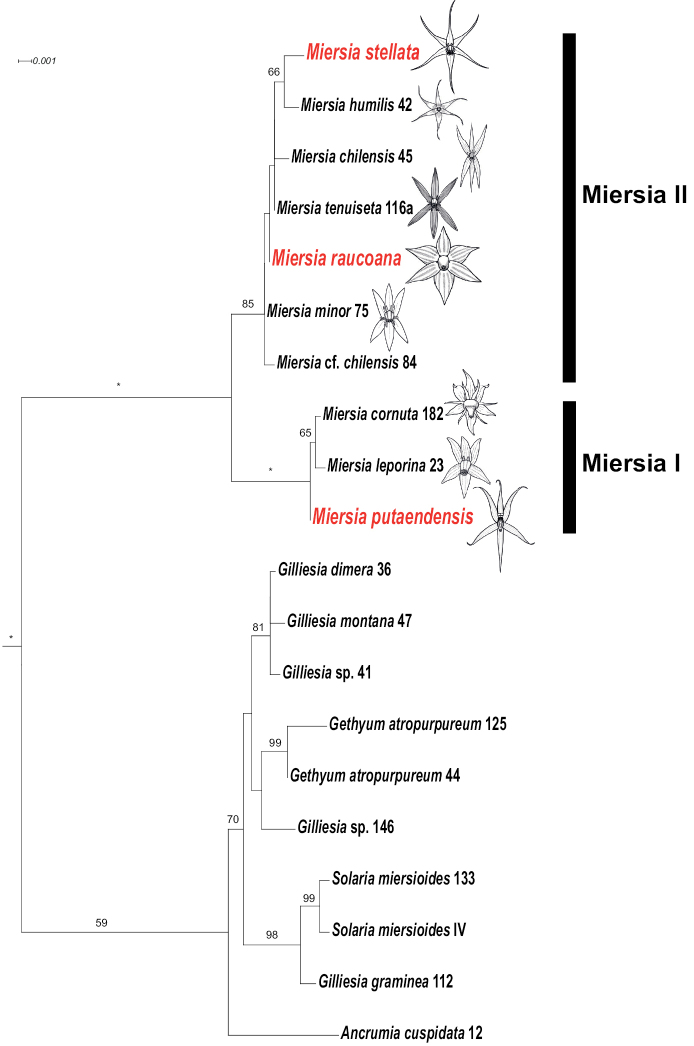
Maximum likelihood phylogram of Gilliesieae based on concatenated analysis of nrDNA ITS and cpDNA (*trnL-F*, *rbcL*). Numbers above branches represent bootstrap (BS) values > 50, asterisks indicate BS = 100. Numbers following species names correspond to accession numbers in Escobar et al. (2020). Novel *Miersia* species being placed within phylogeny are in red font. Two major *Miersia* clades (I, II) are denoted with black bars. Outgroups have been excluded from the figure and root branch is not to scale. Illustrations by Arón Cádiz-Véliz.

Our ML tree is overall congruent with the phylogeny reported in Escobar et al. (2020), and in particular, regarding the monophyly of Gilliesieae and the presence of two major clades: 1) *Gilliesia* + *Ancrumia* + *Gethyum* + *Solaria*, and 2) *Miersia* + *Speea* (= *Miersiahumilis*) (Fig. 2). With respect to relationships within the *Miersia* clade, our phylogeny also agrees with Escobar et al. (2020) regarding the inference of two subclades: 1) *M.cornuta* + *M.leporina* (= Miersia I clade, Fig. 2), and 2) *M.chilensis* + *M.humilis* + *M.minor* + *M.tenuiseta* (= Miersia II clade, Fig. 2). *Miersiaputaendensis* is retrieved with strong support (BS = 100) in the Miersia I clade, whereas *M.raucoana* and *M.stellata* are part of the Miersia II clade, albeit this clade has moderate support (BS = 85; see Fig. 2).

### ﻿Taxonomic treatment

#### ﻿ Miersia stellata

Taxon classificationPlantaeAsparagalesAmaryllidaceae

C.Cuevas & Nic.García sp. nov.

885821A2-BD40-5155-BFAA-7FFE2E0558DF

urn:lsid:ipni.org:names:77306995-1

Figs 3 , 4

##### Diagnosis.

*Miersiastellata* differs from *Miersiahumilis* (Phil.) M.F.Fay & Christenh. by a capitate stigma (vs. trilobed stigma), six bifid, rarely trifid, flat tepaliferous appendages (vs. tepaliferous appendages absent), and a cylindrical to urceolate staminal tube with a short apical reflexed rim (vs. staminal filaments fused in their basal half and covering the ovary, but not forming an urceolate tube).

##### Type.

Chile. Región Metropolitana de Santiago: Provincia de Chacabuco, Comuna de Lampa, cerro Quilhuica, 600 m a.s.l., 17 June 2021, *N. García & C. Cuevas 6132* (holotype: EIF 14823; isotypes: CONC, JBN, SGO).

##### Description.

Terrestrial saxicolous herbs. ***Bulbs*** ovoid, usually flattened due to growth in rock crevices, external cataphylls light brown, 11–15 × 5–10 mm. ***Leaves*** 2–3, linear, hanging, 7–20 × 0.09–0.2 cm. ***Scapes*** 1–2, cylindrical, hollow, 20–70 × 1–1.3 mm. ***Spathe*** 2-valvate, herbaceous, lanceolate, 7–12 × 1.5–2 mm, fused on their basal ¼ (~2.5 mm), whitish with veins inconspicuous or purple spotted. ***Inflorescences*** a pseudo-umbel with 1–2 (–3) slightly zygomorphic, star-shaped flowers; ***pedicels*** unequal, 1.4–2.7 cm long, apex curved in a right angle (~90°). ***Tepals*** 6, free, membranous, light green, rarely purplish, lanceolate, caudate, straight, ***outer*** 12 × 2–2.5 mm, 5 acrodromous veins, ***inner*** 11–11.5 × 1.5–1.8 mm, 3 acrodromous veins, on both whorls only the central is well marked and runs throughout the complete length, cauda 0.4–0.5 mm wide comprising ~2/3 of the tepal’s length. ***Tepaliferous appendages*** 6, green, deeply bifid, rarely trifid, flat, upper pair with lanceolate segments, each segment sometimes shortly bifid, fused at base ~0.6 mm, 2.0–2.5 × 0.4–0.5 mm, lateral appendages one pair on each side, with linear to linear-lanceolate segments, attached to the base of inner tepals, segments fused at base ~0.1 mm long, 2.0–2.5 × ca. 0.2 mm. ***Stamens*** 6, filaments 0.2–0.3 mm long, adnate internally to the staminal tube; ***staminal tube*** cylindrical to urceolate, whitish with two purple longitudinal stripes and three longitudinal folds on its upper side, single longitudinal fold on the lower side, apex with a short reflexed rim, papillose, 2.0–2.5 × 1.5–2.5 mm; ***anthers*** yellow (purple when dry), 0.8–1.0 mm long, exerted. ***Ovary*** superior, spherical to obovoid, 1.0–1.3 mm long, trilocular, 12 ovules per locule, biseriate; ***style*** nodding, exerted, 1.7–2.0 mm long; ***stigma*** capitate. ***Capsules*** obovoid to spherical, 3-valved, 4–8 × 4–6 mm. ***Seeds*** not seen.

##### Distribution and habitat.

*Miersiastellata* has been recorded in a single rocky outcrop in the Quilhuica hill, Lampa (~33.3° S), which is an isolated hill, between the main coastal mountain range (*cordillera de la Costa*) and the basin of Santiago. This south-facing rocky outcrop is at the bottom of a creek at 600 m a.s.l. The new species grows exclusively in rock crevices along with *Tristagmagraminifolium* (Phil.) Ravenna. The surrounding vegetation corresponds to a degraded sclerophyllous arborescent scrub composed of *Lithraeacaustica* (Molina) Hook. & Arn., *Quillajasaponaria* Molina and *Porlieriachilensis* I.M.Johnst.

##### Phenology.

This species has been seen in flowers between May and August. Immature fruits have been recorded during August and September; in general, fructification is low in the population.

##### Etymology.

The specific epithet *stellata* refers to star-shaped form of flowers.

##### Vernacular name.

Although no popular common name is known for *Miersiastellata*, we propose to name it “*estrella de Lampa*” or “Lampa star”.

##### Conservation status.

*Miersiastellata* can be considered Critically Endangered (CR) according to criteria B2ab(iii). Its area of occupancy is < 10 km^2^, with an estimate of 120 m^2^ (~0.0001 km^2^). Only a single population of < 100 individuals has been recorded despite sampling efforts in surrounding areas in suitable seasons and habitats. In addition, it inhabits an area intensely degraded by human activities and is close to a highway with heavy traffic and to populated locations (Batuco, Lampa).

**Figure 3.**
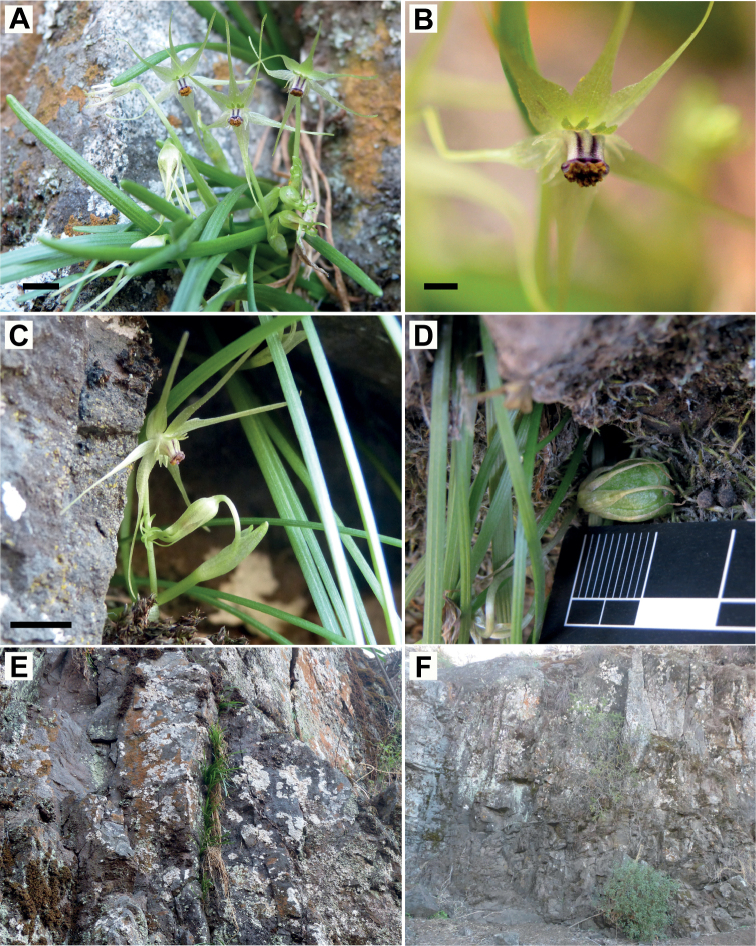
*Miersiastellata* C.Cuevas & Nic.García **A** front view of flowers **B** detail of flower showing tepaliferous appendages and staminal tube **C** lateral view of flower **D** immature fruit **E** habit **F** habitat. Scale bars: 5 mm (**A, C**); 2 mm (**B**). Photos by Nicolás García (**A, C, E, F**), Nicolás Villaseca (**B**), Claudia Cuevas (**D**).

**Figure 4.**
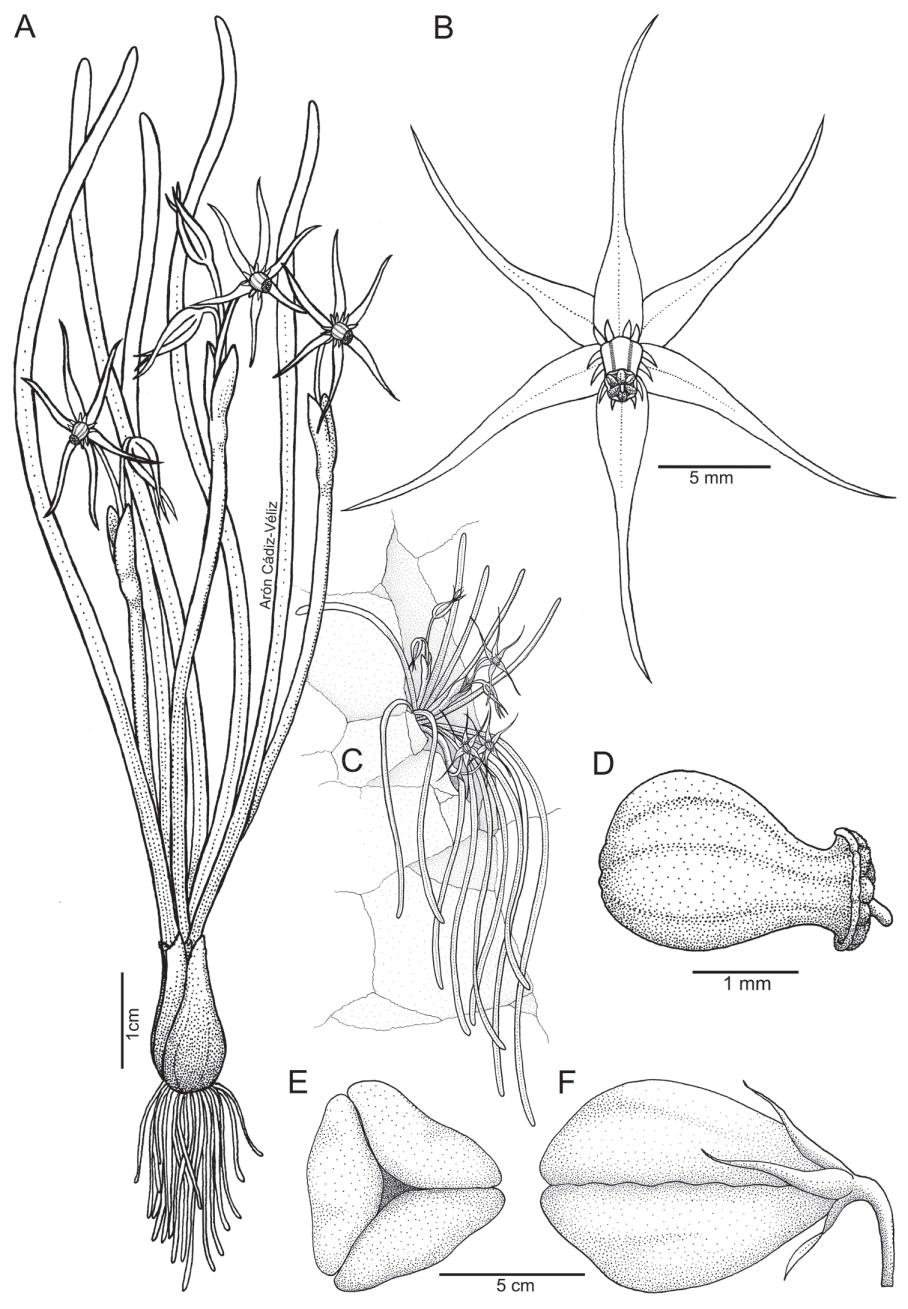
*Miersiastellata* C.Cuevas & Nic.García **A** habit **B** flower (frontal view) **C** plant growing in its natural habitat **D** staminal tube (lateral view) **E** fruit (apical view) **F** fruit (lateral view). Illustration by Arón Cádiz-Véliz.

##### Additional specimens examined

**(*paratypes*). Chile. Región Metropolitana de Santiago**: Provincia de Chacabuco, Comuna de Lampa, cerro Quilhuica, 600 m a.s.l., 5 August 2020, *C. Cuevas s.n.* (EIF); 16 September 2020, *N. García, C. Cuevas & M. Villalobos 5843* (EIF).

#### ﻿ Miersia raucoana

Taxon classificationPlantaeAsparagalesAmaryllidaceae

J.E.Sepúlveda & Nic.García sp. nov.

E7BAE5D0-F404-50A3-8050-F61FC01FEB2E

urn:lsid:ipni.org:names:77306996-1

Figs 5 , 6

##### Diagnosis.

*Miersiaraucoana* differs from *Miersiatenuiseta* Ravenna by the lack of tepaliferous appendages or tiny and awl-shaped, < 0.5 mm long, if present (vs. 6 conspicuous filiform, bifid tepaliferous appendages), a slightly zygomorphic conical staminal tube (vs. strongly zygomorphic urceolate staminal tube), and 3–5 purple longitudinal stripes in each tepal (vs. none or single broad central longitudinal stripe).

##### Type.

Chile. Región del Maule: Provincia de Curicó, Comuna de Rauco, quebrada Guayacán, 535 m a.s.l., 6 July 2021, *N. García, J. Sepúlveda, A. Cádiz-Véliz, C. Soto & M. Tobar 6139* (holotype: EIF 14824; isotypes: CONC, JBN, SGO).

##### Description.

Terrestrial saxicolous herbs. ***Bulbs*** subglobose to ovoid, 10–12 × 7–10 mm, external cataphylls light brown. ***Leaves*** 3–4, linear, 8–23 × 0.08–0.15 cm. ***Scapes*** 2–3, cylindrical, hollow, 10–40 × ca. 0.8 mm. ***Spathe*** 2-valvate, herbaceous, lanceolate, 10–10.5 × 3–3.5 mm, fused on their basal ¼ (~2.5 mm), veins purple. ***Inflorescences*** a pseudo-umbel with 2–5 slightly zygomorphic flowers; ***pedicels*** unequal, 1.5–2.0 cm long. ***Tepals*** 6, free, membranous, creamy white to yellowish (in dry specimens) with 3–5 purple longitudinal stripes each (exceptionally without stripes), lanceolate to obovate, acute, straight to slightly reflexed apically, ***outer*** 8–9 × 3.5 mm, ***inner*** 7–8 × 3.0–3.5 mm. ***Tepaliferous appendages*** absent or inconspicuous, awl-shaped, purple, < 0.5 mm long. ***Stamens*** 6, filaments 0.5–0.8 mm long, diminishing in length towards the downward side of the flower, adnate internally to the staminal tube; ***staminal tube*** conical, apex narrowly tubular (ca. 0.8 × 0.5 mm), slightly zygomorphic, purple, papillose, 3–4 × 2.5–3.0 mm; ***anthers*** 6, yellow (purple when dry), ca. 0.3–0.4 mm long, exerted. ***Ovary*** superior, spherical to obovoid, 1.0–1.5 mm long, trilocular, 10 ovules per locule, biseriate; ***style*** straight to ascending, 1.5–2.0 mm long, reaching the anthers or exerted in mature flowers; ***stigma*** capitate. ***Capsules*** obovoid to spherical, 3-valved, 8 × 6.5 mm, 13–14. ***Seeds*** obovoid, 1.6–2.0 × 1.0–1.4 mm, testa reticulate, 13–14 per capsule.

##### Distribution and habitat.

*Miersiaraucoana* was originally recorded in a rocky east- to northeast-facing slope in the coastal mountain range of Rauco (~34.9°S), Maule Region. During the review process of this article, it was also recorded around the La Palmilla dam, located ca. 3 km north of the typical locality. It can be found growing in rock crevices or in the base of rocky outcrops, between 240 and 540 m a.s.l. The surrounding vegetation corresponds to a sclerophyllous arborescent scrub, where the most abundant species are *Lithraeacaustica* (Molina) Hook. & Arn, *Peumusboldus* Molina, *Vachelliacaven* (Molina) Seigler & Ebinger, *Retanillatrinervia* Hook. & Arn., *Chusqueacumingii* Nees, and *Leucostelechiloensis* (Colla) Schlumpb.

##### Phenology.

*Miersiaraucoana* has been recorded in flowers from May to early August. Fruits have been recorded in late July and throughout August.

##### Etymology.

The specific epithet refers to Rauco, a municipality located to the west of the city of Curicó in the Maule region of Chile.

##### Vernacular name.

We propose to name this species as “*miersia de Rauco*”.

##### Conservation status.

*Miersiaraucoana* can be considered Critically Endangered (CR) under criteria B2ab(iii), because its area of occupancy is < 10 km^2^, with an estimated 1.8 km^2^. Only a single population scattered throughout the latter area, with < 1,000 mature individuals, has been recorded despite sampling efforts in surrounding areas in suitable seasons and habitats. In addition, the area is at risk of forest fires and is subject to land use change for agricultural crops, motorized sporting activities, and goat and cattle ranching.

**Figure 5.**
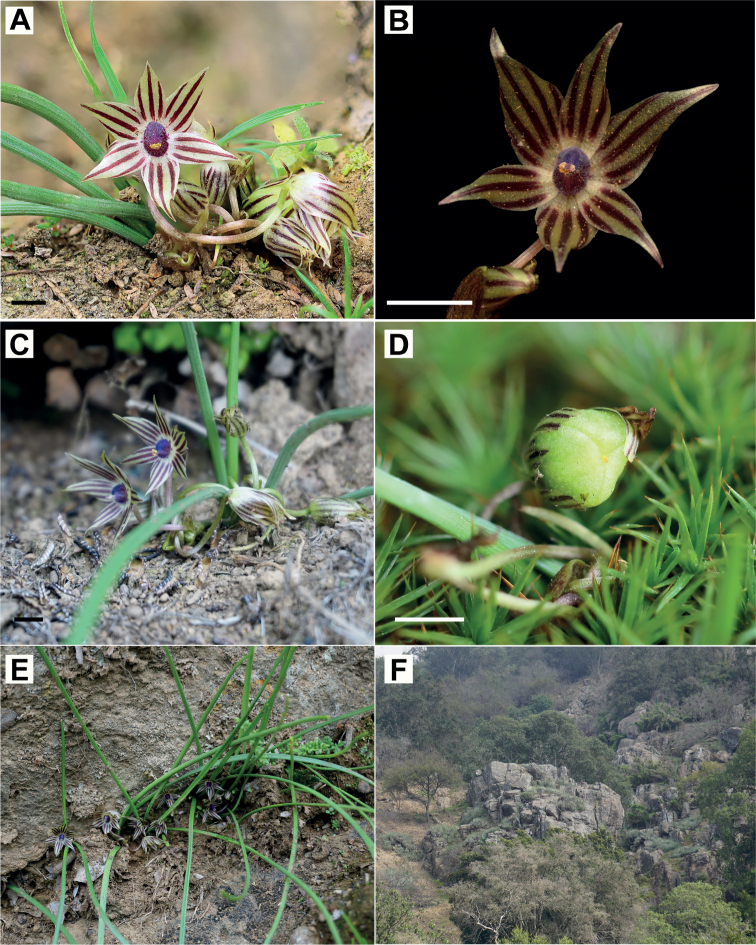
*Miersiaraucoana* J.E.Sepúlveda & Nic.García **A** front view of flower **B** detail of flower lacking appendages **C** lateral view of flowers showing tiny tepaliferous appendages **D** inmature fruit **E** habit **F** habitat. Scale bars: 5 mm. Photos by José Luis Inostroza (**A, D**), Matías Tobar (**B**), Joaquín Sepúlveda (**C, E, F**).

**Figure 6.**
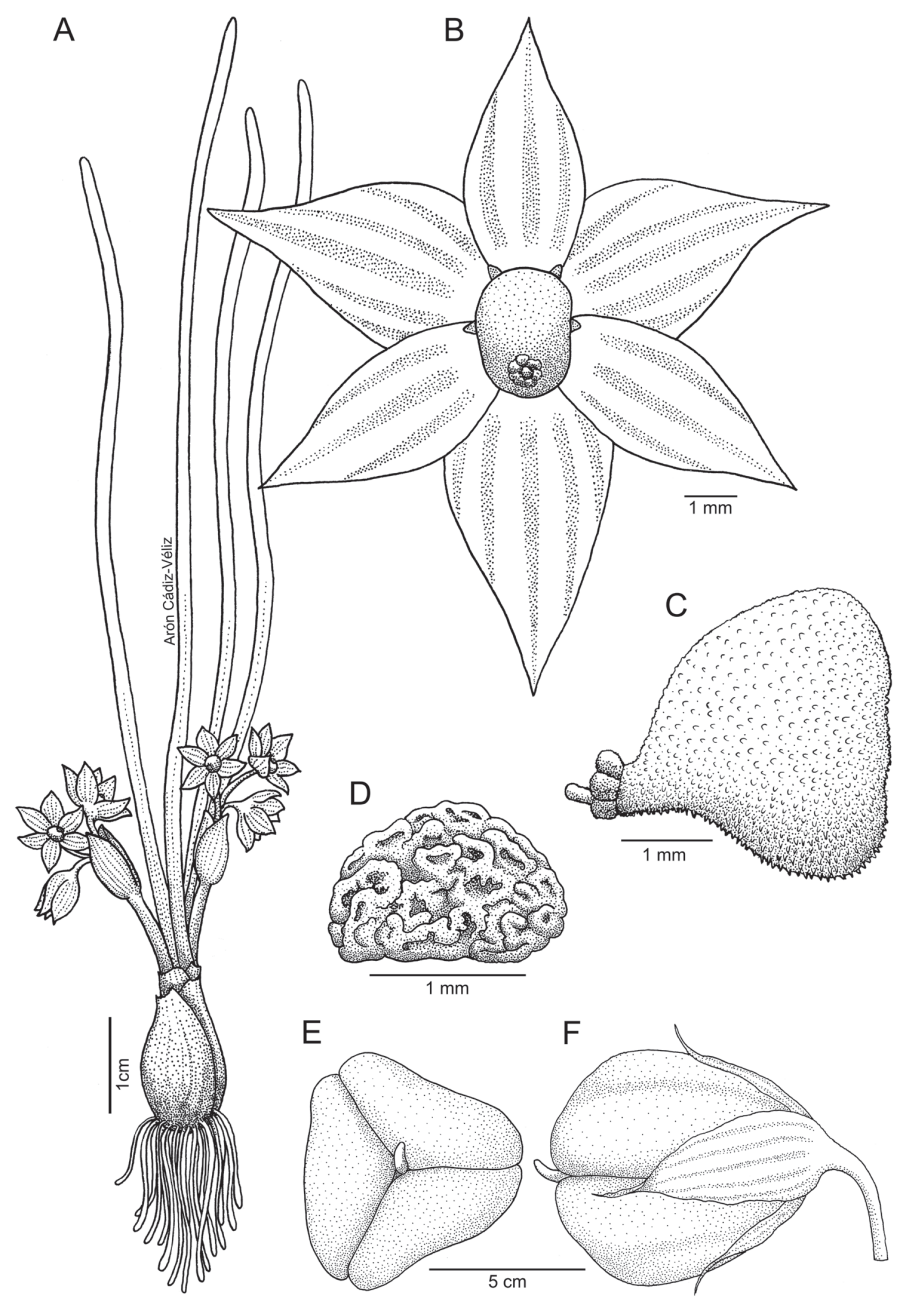
*Miersiaraucoana* J.E.Sepúlveda & Nic.García **A** habit **B** flower (frontal view) **C** staminal tube (lateral view) **D** seed **E** fruit (apical view) **F** fruit (lateral view). Illustration by Arón Cádiz-Véliz.

##### Additional specimens examined

**(*paratypes*). CHILE. Región del Maule**: Provincia de Curicó, Comuna de Rauco, quebrada Guayacán, 535 m a.s.l., 12 August 2020, *J. Sepúlveda s.n.* (EIF).

### ﻿Key to the species of *Miersia* [modified from Cádiz-Véliz (2021)]

**Table:** 

1	Flowers with 2 tepaliferous appendages above the staminal tube	**2**
–	Flowers with 6 tepaliferous appendages around the staminal tube or appendages absent	**3**
2	Tepaliferous appendages lorate to cuneiform, apex truncate, erose and deflected, oriented frontward; white staminal tube featuring an elongated frontal lobe with a purple apical spot	***M.putaendensis***
–	Tepaliferous appendages oblong to subulate, apex entire, obtuse and straight, oriented upward; bluish-green staminal tube with an erect, short, upper lobe without a purple spot	***M.leporina***
3	Tepals clearly reflexed on their distal half. Staminal tube with a globose deflected base. Tepaliferous appendages entire and filiform to narrowly lanceolate	***M.cornuta***
–	Tepals generally straight throughout or slightly reflexed. Staminal tube not globose at base. Tepaliferous appendages divided or absent	**4**
4	Tepals caudate over 2/3 of their length, longer than 11 mm	**5**
–	Tepals acute to acuminate, shorter than 11 mm	**6**
5	Tepaliferous appendages absent. Staminal tube without an apical reflexed rim. Filaments born apically, conspicuous and seeming a continuation of the tube. Stigma trilobed	***M.humilis* (= *Speeahumilis*)**
–	Tepaliferous appendages present. Staminal tube with a short apical reflexed rim. Filaments inserted and born laterally on the inner face of the tube. Stigma capitate	***M.stellata***
6	Tepals creamy white with 2 to 3 purple longitudinal stripes, rarely plain creamy white, perigone actinomorphic. Tepaliferous appendages absent or awl-shaped and shorter than 0.5 mm. Staminal tube conical, purplish; opening central and pointing towards the front of the flower	***M.raucoana***
–	Tepals plain light green to purplish or sometimes with a single central and broad purple longitudinal stripe (in *M.tenuiseta*), perigone zygomorphic. Tepaliferous appendages filiform or flat, bifid to trifid, longer than 0.5 mm. Staminal tube urceolate, whitish to greenish or with a wide purple stripe on upper face; opening lateral, placed towards the lower side of the flower	**7**
7	Tepals acuminate, apex generally reflexed	***M.chilensis***
–	Tepals acute, apex straight or inflexed	**8**
8	Outer tepals lanceolate to linear-lanceolate. Appendages filiform, upper and lateral similar	***M.tenuiseta***
–	Outer tepals ovate to oval-lanceolate. Appendages flat, upper and lateral different	***M.minor***

## ﻿Discussion

The present phylogenetic analysis of tribe Gilliesieae coincides with the results of Escobar et al. (2020), which was expected considering that we used the same molecular markers and some of the sequences produced by that work. Therefore, we also identified the same taxonomic issues, for instance, the paraphyly of *Gilliesia* with respect to *Solaria* and *Gethyum*, and the putative sister relationship of *Ancrumia* to the rest of that clade. On the other hand, the single species in *Speea* (i.e., *Speeahumilis*) is well embedded within the *Miersia* clade (Fig. 2), with which it shares several putative synapomorphies, including oblong capsules, 12 chromosomes and filaments fused at least covering the ovary (Escobar et al. 2020); therefore, this species has been treated as *Miersiahumilis* (Phil.) M.F.Fay & Christenh. in the present work. A generic circumscription of *Miersia* including *Speea* renders the former genus monophyletic according to currently available phylogenetic information. In this sense, *Miersia* s.l. is composed of nine species and is well diagnosed in the context of Gilliesieae taxonomy by the presence of six functional stamens and the lack of staminodes (Escobar et al. 2020).

Despite low resolution within the *Miersia* subclades, the phylogenetic position of the recently described species is well supported within Miersia I in the case of *M.putaendensis* and within Miersia II for *M.raucoana* and *M.stellata*. Given these subclade circumscriptions, Miersia I is composed of three species that have the northernmost distributions within the genus between the basins of the Choapa and Aconcagua rivers (~32°-33°S; Escobar 2012; Cádiz-Véliz 2021). Separate analyses of the nrITS and cpDNA datasets show a single strong cytonuclear discordance in *Miersia* regarding the position of *M.putaendensis* within Miersia I (Suppl. materials 1, 2: Figs S1 and S2, also available in Zenodo, doi: 10.5281/zenodo.6581791), which could be indicating a hybrid origin for one of the species involved in this clade. This mechanism of speciation was suggested as putatively present within *Miersia* by Escobar et al. (2020). No clear synapomorphy or diagnostic character has been detected for this subclade; however, it is noteworthy to mention that the only two species with only two upper appendages in *Miersia*, *M.leporina* Ravenna and *M.putaendensis*, belong to this group.

On the other hand, Miersia II contains at least six species with their distributions concentrated between the Maipo and Maule river basins (~33–36°S; Escobar 2012). However, the consistently low phylogenetic resolution and short branches in the Miersia II clade make it impossible to confidently ascertain relationships among species given the available data and suggest a rapid diversification within this clade (Escobar et al. 2020). As in Miersia I, no synapomorphy or diagnostic character has been suggested for this clade (Escobar et al. 2020), which in turn shows considerable floral variation and diversification, exemplified by the outstanding divergence of *M.humilis* in characters such as stigma type and degree of fusion of staminal filaments, which otherwise are constant within *Miersia*.

Generic recircumscriptions within the *Gilliesia* clade are also desirable to comply with the primary principle of monophyly given a phylogenetic approach to biological classification (e.g., Wiley and Lieberman 2011; Judd et al. 2016); however, this issue is out of the scope of the present work. A comprehensive evolutionary study and proposal of generic classification for Gilliesieae must also include sequences and morphological considerations for *Trichlora* from Peru and *Schickendantziella* from Argentina and Bolivia, which are the only non-Chilean taxa in the tribe and have not been included in previous molecular phylogenetic research of the group (Escobar et al. 2020). Future studies should also include data from multiple low-copy nuclear genes to clarify the phylogeny of Gilliesieae, providing a robust framework to reassess the generic taxonomy and evaluate the diversification of this clade.

## Supplementary Material

XML Treatment for Miersia stellata

XML Treatment for Miersia raucoana

## Supplementary materials

Supplementary material 1 Figure S1 Data type Eps file. Explanation note Maximum likelihood cladogram of Gilliesieae and outgroups based on nrDNA ITS. Numbers above branches represent bootstrap (BS) values > 50, asterisks indicate BS = 100. Numbers following species names correspond to accession numbers in Escobar et al. (2020). https://binary.pensoft.net/file/760115This dataset is made available under the Open Database License (http://opendatacommons.org/licenses/odbl/1.0/). The Open Database License (ODbL) is a license agreement intended to allow users to freely share, modify, and use this Dataset while maintaining this same freedom for others, provided that the original source and author(s) are credited.Nicolás García, Claudia Cuevas, Joaquín E. Sepúlveda, Arón Cádiz-Véliz, María José Román

Supplementary material 2 Figure S2 Data type Eps file. Explanation note Maximum likelihood cladogram of Gilliesieae and outgroups based on concatenated analysis of cpDNA (*trnL-F*, *rbcL*). Numbers above branches represent bootstrap (BS) values > 50, asterisks indicate BS = 100. Numbers following species names correspond to accession numbers in Escobar et al. (2020). https://binary.pensoft.net/file/760116This dataset is made available under the Open Database License (http://opendatacommons.org/licenses/odbl/1.0/). The Open Database License (ODbL) is a license agreement intended to allow users to freely share, modify, and use this Dataset while maintaining this same freedom for others, provided that the original source and author(s) are credited.Nicolás García, Claudia Cuevas, Joaquín E. Sepúlveda, Arón Cádiz-Véliz, María José Román

Supplementary material 3 Table S1 Data type Docx file. Explanation note **Table S1.** GenBank accession numbers of DNA sequences used in this study. Previously unpublished sequences are denoted with an asterisk (*). https://binary.pensoft.net/file/760117This dataset is made available under the Open Database License (http://opendatacommons.org/licenses/odbl/1.0/). The Open Database License (ODbL) is a license agreement intended to allow users to freely share, modify, and use this Dataset while maintaining this same freedom for others, provided that the original source and author(s) are credited.Nicolás García, Claudia Cuevas, Joaquín E. Sepúlveda, Arón Cádiz-Véliz, María José Román
