# ﻿Corrigendum: García N, Cuevas C, Sepúlveda JE, Cádiz-Véliz A, Román MJ (2022) Two new species of *Miersia* and their phylogenetic placements alongside the recently described *M.putaendensis* (Gilliesieae, Allioideae, Amaryllidaceae). PhytoKeys 211: 107–124. doi: 10.3897/phytokeys.211.87842

**DOI:** 10.3897/phytokeys.214.97612

**Published:** 2022-11-30

**Authors:** Nicolás García, Claudia Cuevas, Joaquín E. Sepúlveda, Arón Cádiz-Véliz, María José Román

**Affiliations:** 1 Herbario EIF & Laboratorio de Evolución y Sistemática, Facultad de Ciencias Forestales y de la Conservación de la Naturaleza, Universidad de Chile, Av. Santa Rosa 11315, La Pintana, Santiago, Chile Universidad de Chile Santiago Chile; 2 Instituto Agroecosistemas, Curicó, Chile Instituto Agroecosistemas Curico Chile; 3 Instituto de Biología, Facultad de Ciencias, Pontificia Universidad Católica de Valparaíso, Campus Curauma, Avenida Universidad 330, Valparaíso, Chile Pontificia Universidad Católica de Valparaíso Valparaíso Chile; 4 Department of Biology, University of Florida, Gainesville, Florida 32611, USA University of Florida Gainesville United States of America

According to Escobar (2012), there are two kinds of floral appendages around the staminal tube or urn in *Miersia*: 1) the upper pair of appendages are of staminal origin and 2) the four lateral appendages, one pair on each side of the staminal tube, are of tepaliferous origin. Consequently, mentions to “tepaliferous appendages” in diagnoses, descriptions, identification key and figure captions of our recent article (García et al. 2022) should be changed to “floral appendages”, to denote the mixed nature of those structures in most species of *Miersia*.
