# Supplementary material for: ﻿Two new species of Miersia and their phylogenetic placements alongside the recently described M.putaendensis (Gilliesieae, Allioideae, Amaryllidaceae)
Source: PhytoKeys. 2022 Oct 20;211:107–24. doi: 10.3897/phytokeys.211.87842 (PMC9836716; doi:10.3897/phytokeys.211.87842)
Supplement: Supplementary material 3 — Table S1 [file phytokeys-211-107_article-87842__-s003.docx]

Table S1. GenBank accession numbers of DNA sequences used in this study. Previously unpublished sequences are denoted with an asterisk (*).

| Species | nrITS | *rbcL* | *trnL-F* |
| --- | --- | --- | --- |
| *Ancrumia cuspidata* Harv. ex Baker 12 | MN818878 | MN841470 | MN841598 |
| *Gethyum atropurpureum* Phil. 44 | MN818872 | MN841466 | MN841571 |
| *Gethyum atropurpureum* Phil. 125 | MN818873 | MN841467 | MN841569 |
| *Gilliesia dimera* Ravenna 36 | MN818879 | MN841471 | MN841591 |
| *Gilliesia graminea* Lindl. 112 | MN818890 | MN841482 | MN841575 |
| *Gilliesia montana* Poepp. & Endl. 47 | MN818894 | MN841483 | MN841580 |
| *Gilliesia* sp. 41 | MN818898 | MN841487 | MN841583 |
| *Gilliesia* sp. 146 | MN818900 | MN841489 | MN841584 |
| *Miersia chilensis* Lindl. 45 | MN818920 | MN841506 | MN841556 |
| *Miersia* cf. *chilensis* Lindl. 84 | MN818931 | MN841511 | MN841547 |
| *Miersia cornuta* Phil. 182 | MN818925 | ----- | MN841562 |
| *Miersia humilis* (Phil.) M.F. Fay & Christenh. 42 | MN818945 | MN841525 | MN841559 |
| *Miersia leporina* Ravenna 23 | MN818927 | MN841508 | MN841565 |
| *Miersia minor* Kunth 75 | MN818918 | MN841504 | MN841546 |
| *Miersia putaendensis* A. Cádiz-Véliz | ON603535* | ON691517* | ON605648* |
| *Miersia raucoana* J.E. Sepúlveda & Nic. García | ON603536* | ON691518* | ON605649* |
| *Miersia stellata* C. Cuevas & Nic. García | ON603534* | ON691516* | ON605647* |
| *Miersia tenuiseta* Ravenna 116a | MN818936 | MN841516 | MN841554 |
| *Solaria miersioides* Phil. 133 | MN818939 | MN841519 | MN841590 |
| *Solaria miersioides* Phil. IV | MN818943 | MN841523 | MN841573 |
| *Ipheion uniflorum* (Lindl.) Raf. | MN818902 | AF116992 | AF117021 |
| *Leucocoryne pauciflora* Phil. | MN818903 | AF116998 | AF116998 |
| *Nothoscordum gracile* (Aiton) Stearn. | MN818938 | MN841518 | MN841600 |
| *Nothoscordum hirtellum* (Kunth) Herter | MN818901 | MN841490 | MN841599 |
| *Tristagma bivalve* (Hook. ex Lindl.) Traub | MN818950 | Z69206 | AF117051 |
| *Tulbaghia capensis* L. | MN818951 | MN841528 | MN841601 |
